# Empowering Dementia Carers With an iSupport Virtual Assistant (e-DiVA) in Asia-Pacific Regional Countries: Protocol for a Pilot Multisite Randomized Controlled Trial

**DOI:** 10.2196/33572

**Published:** 2021-11-16

**Authors:** Tuan Anh Nguyen, Kham Tran, Adrian Esterman, Bianca Brijnath, Lily Dongxia Xiao, Penelope Schofield, Sunil Bhar, Nilmini Wickramasinghe, Ronald Sinclair, Thu Ha Dang, Sarah Cullum, Yuda Turana, Ladson Hinton, Katrin Seeher, Andre Q Andrade, Maria Crotty, Susan Kurrle, Stefanie Freel, Thang Pham, Thanh Binh Nguyen, Henry Brodaty

**Affiliations:** 1 Social Gerontology Division National Ageing Research Institute Melbourne Australia; 2 UniSA Clinical and Health Sciences University of South Australia Adelaide Australia; 3 Health Strategy and Policy Institute Ministry of Health Hanoi Vietnam; 4 College of Nursing and Health Sciences Flinders University Adelaide Australia; 5 Department of Psychology, School of Health Sciences Swinburne University of Technology Melbourne Australia; 6 Faculty of Sciences University of Adelaide Adelaide Australia; 7 Faculty of Medical and Health Sciences University of Auckland Auckland New Zealand; 8 School of Medicine Atma Jaya Catholic University of Indonesia Jakarta Indonesia; 9 Department of Psychiatry and Behavioral Sciences University of California, Davis Sacramento, CA United States; 10 Department of Mental Health and Substance Use World Health Organization Geneva Switzerland; 11 Quality Use of Medicines and Pharmacy Research Centre University of South Australia Adelaide Australia; 12 College of Medicine and Public Health Flinders University Adelaide Australia; 13 Faculty of Medicine and Health University of Sydney Sydney Australia; 14 Department of Germanic Languages and Literature University of Toronto Toronto, ON Canada; 15 Department of Neurology and Alzheimer Disease Vietnam National Geriatric Hospital Hanoi Vietnam; 16 Centre for Healthy Brain Ageing School of Psychiatry University of New South Wales Sydney Australia

**Keywords:** Dementia, informal carer, iSupport, virtual assistant, digital health

## Abstract

**Background:**

Dementia is a global public health priority with an estimated prevalence of 150 million by 2050, nearly two-thirds of whom will live in the Asia-Pacific region. Dementia creates significant care needs for people with the disease, their families, and carers. iSupport is a self-help platform developed by the World Health Organization (WHO) to provide education, skills training, and support to dementia carers. It has been adapted in some contexts (Australia, India, the Netherlands, and Portugal). Carers using the existing adapted versions have identified the need to have a more user-friendly version that enables them to identify solutions for immediate problems quickly in real time. The iSupport virtual assistant (iSupport VA) is being developed to address this gap and will be evaluated in a randomized controlled trial (RCT).

**Objective:**

This paper reports the protocol of a pilot RCT evaluating the iSupport VA.

**Methods:**

Seven versions of iSupport VA will be evaluated in Australia, Indonesia, New Zealand, and Vietnam in a pilot RCT. Feasibility, acceptability, intention to use, and preliminary impact on carer-perceived stress of the iSupport VA intervention will be assessed.

**Results:**

This study was funded by the e-ASIA Joint Research Program in November 2020. From January to July 2023, we will enroll 140 dementia carers (20 carers per iSupport VA version) for the pilot RCT. The study has been approved by the Human Research Committee, University of South Australia, Australia (203455).

**Conclusions:**

This protocol outlines how a technologically enhanced version of the WHO iSupport program—the iSupport VA—will be evaluated. The findings from this intervention study will provide evidence on the feasibility and acceptability of the iSupport VA intervention, which will be the basis for conducting a full RCT to assess the effectiveness of the iSupport VA. The study will be an important reference for countries planning to adapt and enhance the WHO iSupport program using digital health solutions.

**Trial Registration:**

Australian New Zealand Clinical Trials Registry ACTRN12621001452886; https://tinyurl.com/afum5tjz

**International Registered Report Identifier (IRRID):**

PRR1-10.2196/33572

## Introduction

Dementia is a global public health priority, and its global prevalence is growing exponentially [1], tripling from 46.8 million in 2015 to approximately 150 million by 2050 [2]. The fastest growth is occurring in the Asia-Pacific region, where 70 million people are estimated to be living with dementia by 2050 [3]. Dementia is among the leading causes of disability and dependency in older people, creating significant care needs for people with dementia, their families, and carers, affecting social, health, well-being, and economic dimensions [1,2]. In 2018, dementia was estimated to cost US $1 trillion worldwide, and the cost is predicted to rise to US $2 trillion by 2030 [2]. The catastrophic costs of long-term care drive many families of people with dementia into poverty, strain health and social systems, and is a significant impost on government budgets [1].

Approximately 60% of people with dementia live in low- and middle-income countries (LMICs) and the costs of care are mostly borne by family members [2]. Health and social care systems in LMICs are often not well-developed or well-funded, resulting in unmet care needs for people with dementia and their carers [3,4]. In high-income countries (HICs), most people with dementia also live at home and receive care from their families [3,5]. Health and social care systems in HICs are often fragmented and unable to meet all the needs of people with dementia, especially people from culturally and linguistically diverse (CALD) groups [6]. For example, care arrangements and family carer input are disproportionately high for CALD people with dementia in Australia [7] and Māori families in New Zealand [6], which result in them experiencing more psychological and economic burdens associated with care.

Provision of unpaid care often exposes family carers to pronounced stress, resulting in mental and physical health deterioration, and loss of productivity and income [1,3]. Women, who provide the bulk of family care, are disproportionately affected [8]. In LMICs where aged care facilities and other formal support services are underdeveloped, such impacts can be devastating [9]. Supporting family carers through low-cost and sustainable nonpharmacological approaches is much needed to avoid potentially dangerous alternatives with limited efficacy, such as prescribing psychotropic medications, hospitalization, and institutionalization**.** Providing practical support for carers of people with dementia is viewed as an essential part of national dementia plans to maintain the health and well-being of people with dementia, reduce costs related to dementia, and relieve burden on the health and social care systems [10].

Emerging web-based training and support programs have shown positive effects on improving dementia carers’ mental health outcomes with considerable potential for scaling up [11]. To provide practical support for carers, the World Health Organization (WHO) developed “iSupport for Dementia,” a self-learning evidence-based web-based skills training and support program for informal dementia carers, which can be adapted for use in different countries [12]. iSupport offers education, skills training, and support for carers, using problem-solving and cognitive behavioral therapy techniques. The web-based program contains 23 lessons within five modules: (1) what is dementia; (2) being a carer; (3) self-care; (4) providing care; and (5) dealing with behavior changes. Each lesson comprises interactive exercises, and carers receive immediate feedback to their answers, as well as certification when lessons are completed.

The discontinuation of services that has resulted from COVID-19 and its impact on carers of people with dementia (eg, increased social isolation and caregiving burden) emphasizes the need for concise, easy-to-read tips for dementia family carers. This need led the WHO to develop iSupport Lite [13]. Carer feedback from an Australian iSupport adaptation study also demonstrated the need for iSupport enhancement with a more user-friendly version, mechanisms for real-time support, and provision of peer support [14]. They also identified the need to adapt iSupport for CALD communities given their different care needs [14]. iSupport has not been adapted for use in most countries in the Asia-Pacific Region (APR). The exception includes India, where study findings underscored the need to pay close attention to cultural adaptations of the program to improve its acceptability and accessibility [15]. However, cultural adaptation of the iSupport program requires resources, which may not be achievable in many LMICs in the APR. International collaboration is much needed to address the resource issues.

A dearth of scientific evidence on effective cultural adaptation is significant impediments for the uptake of iSupport in many APR countries grappling with the public health challenges of dementia. The e-ASIA funded e-DiVA project addresses these gaps by (1) developing and evaluating an iSupport virtual assistant (VA) to support dementia carers through a partnership among four APR countries: Australia, Indonesia, New Zealand, and Vietnam; and (2) building capacity of researchers and nongovernment organization (NGO) partners to support this development and evaluation. This paper reports the protocol of a pilot randomized control trial (RCT) evaluating the iSupport VA.

## Methods

### Methodological Approaches

This study will operationalize the UK Medical Research Council guidelines for developing and evaluating complex interventions and will focus on the first two stages of this guide: (1) development and (2) feasibility and piloting [16]. A co-design approach will be applied to involve carers in the design process, focusing on the challenges and issues from their perspective and designing solutions to enhance desirability, acceptability, and usability of the intervention [17]. This work will culminate in the development of the iSupport VA comprising a website and a smart device app that will allow carers to search topics of their choice using text or voice commands and provide video instruction to support them in their caring role. Feasibility, acceptability, intention to use, and preliminary impact on carer perceived stress of the iSupport VA will be conducted via a pilot RCT using established approaches [18,19].

### Theoretical Approach

Guided by the Stress/Health model [20], this study proposes to address the identified gaps through a 3-year research program, which uses established theoretical and methodological approaches to culturally and contextually adapt WHO iSupport for use in the aforementioned 4 countries [21]. The Stress/Health model provides a theoretical framework for understanding multidimensional stressors (primary stressors: care recipients’ changed behavior and disability; secondary stressors: family conflict and work difficulty) and adaptive capacities (coping and social support) that collectively affect carer mental health or well-being [22]. iSupport is a multi-component intervention that builds carer skills for managing different stressors and improving adaptive capacities to improve carer mental health and well-being.

### Study Design

**Figure 1:**
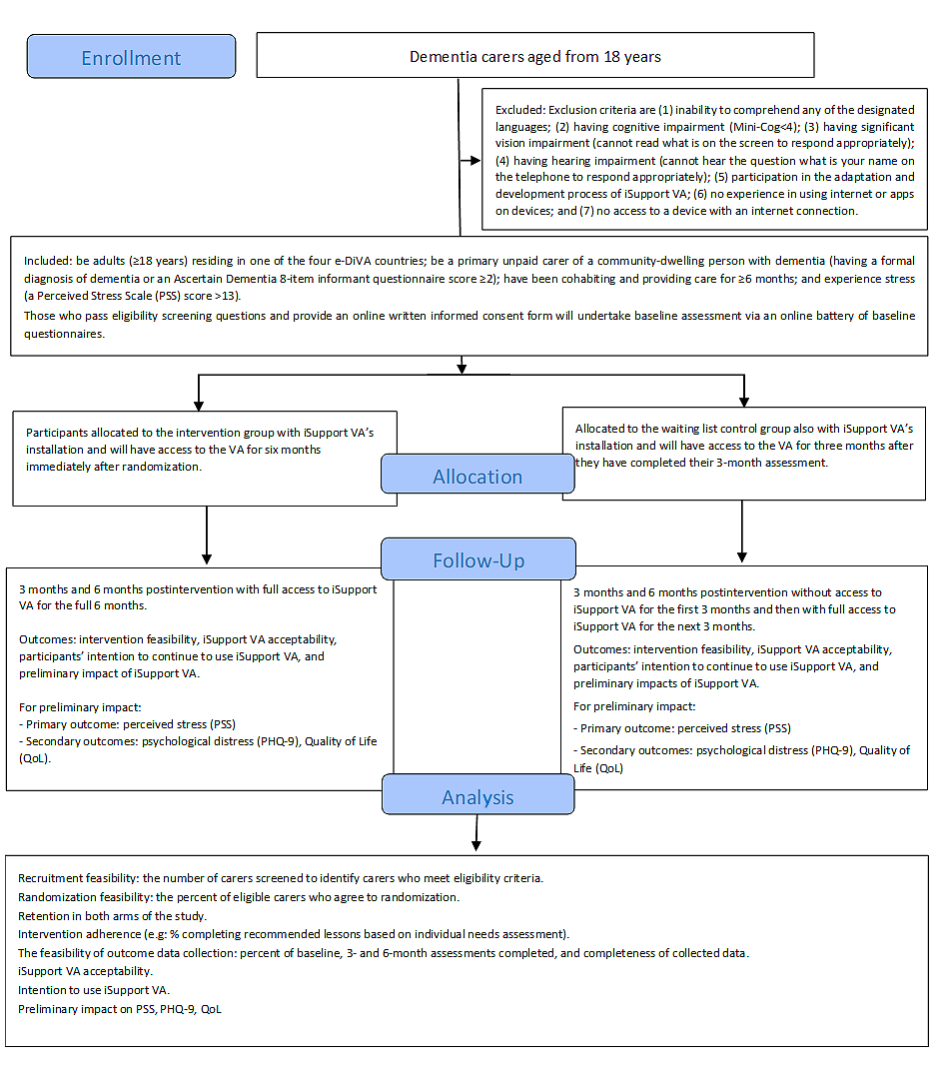
Flow diagram of the pilot randomized controlled trial. PHQ-9: Patient Health Questionnaire, 9-item; VA: virtual assistant.

A pilot, waitlist RCT will be used with 140 carers (20 carers for each of the 7 versions of iSupport VA (English, Bahasa, and Vietnamese in Australia; Vietnamese in Vietnam; Bahasa in Indonesia; and English and Maori in New Zealand). Carers will be randomized into (1) an intervention group (10 carers per version of iSupport VA) that will use the iSupport VA for 6 months immediately following randomization, or (2) a waiting list control group (10 carers/version) that will receive access to the iSupport VA 3 months after randomization (Figure 1). While pilot studies are not powered to examine effectiveness, this pilot RCT will provide data about acceptability, useability, and preliminary impact of the intervention, as well as the feasibility of the methods and procedures [18] for a fully powered RCT to test effectiveness.

#### Inclusion Criteria

Participants eligible for the pilot RCT study will (1) be adults (≥18 years) residing in one of the aforementioned 4 countries (2) be a primary unpaid carer of a community-dwelling person with dementia (having a formal diagnosis of dementia as reported by the carer or an Ascertain Dementia 8-item Informant Questionnaires (AD-8) score of ≥2) [23], (3) have been cohabiting and providing care for ≥6 months, and (4) experience stress (a Perceived Stress Scale [PSS] score >13) [24].

#### Exclusion Criteria

Exclusion criteria are (1) inability to comprehend any of the designated languages, (2) having cognitive impairment (Mini-Cog < 4) [25], (3) having significant vision impairment (cannot read what is on the screen to respond appropriately), (4) having hearing impairment (cannot hear the question “What is your name?” on the telephone to respond appropriately), (5) participation in the adaptation and development process of iSupport VA, (6) having no experience in using the internet or apps on devices, and (7) having no access to a device with an internet connection.

#### Recruitment Procedure

Multiple recruitment strategies will be used. Our local NGO partners (eg, Dementia Australia [DA], Alzheimer’s Indonesia [ALZI], and Alzheimers New Zealand) and local clinics and hospitals will announce the study on their website, social media pages, and newsletter. In Australia, study announcements will also be placed in memory and geriatric clinics managed by the Southern Adelaide Local Health Network, and Resthaven South Australia, the Step-Up for Dementia Research group in New South Wales and Western Australia, and the National Ageing Research Institute (NARI)’s CALD Research Engagement Network, the latter hosts a database of >1000 CALD community groups across Australia. We will leave brochures and posters in hospitals, clinics, senior centers that collaborate with ALZI in Indonesia, dementia services at local District Health Boards in New Zealand, and memory clinics and dementia units in hospitals in Vietnam. Treating clinicians of people with dementia will advise carers about the study during their consultations. All study announcements will refer to our e-DiVA project website, where carers interested in the study can register to participate. Potential participants will be contacted by telephone and email or text message via mobile phones and will receive study information and eligibility screening questions, and will be directed to the web-based written informed consent form. Carers who fulfil eligibility criteria and provide consent will receive a weblink to the internet-based battery of baseline questionnaires and will then be included in the study.

### Randomization Procedure

After enrollment, carers will be randomized separately for each iSupport VA version to either intervention or waiting list control groups (1:1 ratio). A permuted block randomization of size 4 will be used to ensure an even balance of carers in each group throughout the study period. A central clinical trials service in Australia will undertake randomization for each iSupport version in the aforementioned 4 countries.

### Interventions

Seven versions of the iSupport VA, comprising a web-based app, will be built from the content of the adapted iSupport. The VA will be designed to make the iSupport program easier and simpler to use on all device types including smart phones, tablets, and laptop and desktop computers. The VA will first ask individual carers in their respective languages to undergo an assessment of their key education and support needs. Using the finding of this initial need assessment, the algorithm of the VA will provide tailored interventions (ie, recommending specific modules or lessons in the iSupport program) to match each individual’s need profiles. The VA will provide text or voice command search options (similar to Google Assistant). The search outcomes will be in text, picture, or video formats with detailed instructions. Users can choose specific modules or any part of the program simply by selecting a specific topic.

Weblinks for existing health and well-being, aged, and social care services for dementia and other important information (eg, first-aid tips, emergency contact numbers, etc) in each country will be incorporated in VA versions. With carers’ consent, the VA will support web-based peer support groups. These peer support groups will enable sharing of experiences, practical tips, and peer support either in real time video or through text chats among logged in members, or through an asynchronous messaging system. The app will have the capability for a “Personal Diary” for users to write their action plans or scheduled medical appointments and will remind users to follow the scheduled plans and appointments.

Carers randomized to the intervention group will receive a weblink to the iSupport VA app together with the intervention login details, and they will have access to the intervention for 6 months immediately after randomization. Those in the waiting list control group will also receive the iSupport VA. However, they will be informed that they will receive login details for iSupport VA after completing the 3-month assessment. After completing their 3-month assessment, all waiting list control carers will have full access to iSupport VA for 3 months, which will increase the number of dementia carers to access the iSupport VA in this pilot RCT. Intervention dose will be defined as the percentage of lessons completed of the total lessons recommended by the iSupport VA at 3 months in the intervention group, based on the initial need assessment. Monthly phone contact with carers will be also conducted to assess their use of the intervention. Notes from these contacts will be recorded. A local research team member fluent in the country’s appropriate language will be available and contactable by telephone or email if carers need technical assistance.

### Outcomes

The intervention feasibility will be evaluated with reference to recruitment, randomization, retention, treatment adherence, and assessment processes [18]. At the end of the RCT, carers in the intervention group (10 per iSupport VA version) will undergo a 30-60–minute semi-structured exit interview to assess iSupport VA acceptability, including most and least relevant and helpful aspects, the main barriers and facilitators of using the VA, and recommended improvements. All 140 carers will be asked to complete a questionnaire to assess their intention to use the iSupport VA. The questionnaire will include one question: “How will you (dementia carer) use the iSupport VA in the coming six months?” Guided by the Unified Theory of Acceptance and Use of Technology (UTAUT) [19], the questionnaire will assess the direct determinants of intention. These are performance expectancy (the degree to which using the iSupport VA will provide benefits in performing caring role), effort expectancy (the degree of ease associated with the use of the VA), and social influence (the degree to which a carer perceives that others vital to them believe that they should use the VA).

Outcomes will be assessed within the iSupport web-based tool using encryption for data protection at baseline (t_0_), post-intervention (3 months after baseline; t_1_), and follow-up (6 months after baseline; t_2_). For measuring the preliminary impact, the primary outcome will be change in perceived stress [24] at t_1_. The secondary outcomes will include change in psychological distress [26] and global Visual Analogue Scale quality of life [27] at t_1_. To account for a potential contamination of the control group owing to the availability of the generic version of WHO iSupport web-based program, at their 3-month assessment, carers in the control group will be asked if they have accessed the WHO iSupport manual or generic web-based program. Where local-language versions are unavailable, study instruments will be translated and back-translated, followed by reconciliation and review by bilingual experts to develop local language versions. The baseline measures will include language version, characteristics of carers (age; sex; ethnicity; educational, occupational and economic attainments; cohabitation status; and relationship with the care recipient), and care recipients (age, sex, dementia duration, type, and severity [28], and behavioral and psychological symptoms [29]). Further analysis will include a comparison of change in outcomes in the 2 groups between t_0_ and t_3_. This will compare long-term use (6 months) of the tool versus short-term (3 months).

### Analyses and Evaluation

Recruitment feasibility will be assessed on the basis of the number of carers screened to identify carers who meet eligibility criteria. Randomization feasibility will be assessed on the basis of the percentage of eligible carers who agree to randomization. Subject retention in both arms of the study will be evaluated. We will also track intervention adherence (eg, the rate of completing lessons recommended by the VA based on the initial carer need assessment). We will assess the feasibility of assessing study outcomes in terms of the percentage of the baseline, 3-, and 6-month assessments completed and the completeness of the collected data. The criteria for determining feasibility success will be as follows: (1) average of 3 carers per iSupport version per month (approximately 140 carers for 7 iSupport VA versions in the aforementioned 4 countries over 7 months) can be recruited; (2) at least 70% of all eligible carers can be recruited; (3) complete baseline and follow-up assessments by at least 70% of all recruited carers. iSupport VA acceptability will be analyzed thematically. The association between the UTAUT construct [19] and the intention to use iSupport VA will be assessed using logistic regression analyses. For preliminary impact assessment, linear mixed-effects modeling will be used to analyze changes in primary and secondary outcomes from baseline to 3 and 6 months. For dichotomous or noncontiguous outcome measures, appropriate generalized linear mixed-effects models will be applied. All analyses will be adjusted for the language version. One chief investigator of the project is a biostatistician, who will support the research and RCT from designing, collecting, analyzing, and presenting the data. A detailed statistical analysis plan will be developed before unbinding the data and any analyses.

### Data Management and Monitoring

The data collection and management procedures have been approved by the human research ethics committee (The University of South Australia, approval number: 203455). All personal information on people with dementia and their carers will be retained in the pseudonymized data set using a unique participant ID, separate from the main data set, and will not be shared; these data will be accessible to only the project leader. All RCT data will be stored securely to protect confidentiality before, during, and after the trial. Data entry, coding, and analysis of the de-identified data will be conducted by trained and experienced researchers.

Any unexpected outcomes or serious adverse events will be discussed by the project executive committee. RCT progress will be updated in monthly meetings by the study’s executive committee and country leaders. The committee will monitor the recruitment, intervention, and any concerns related to the study.

### Ethics, Dissemination, and Protocol Amendment

#### Ethics

The study has been approved by the Human Research Ethics Committee (HREC) of the University of South Australia. Each study site also seeks their own local IRB approval. Informed consent material is available in local languages of Vietnamese, Bahasa, and Maori with the approved protocol.

#### Dissemination and Protocol Amendment

The primary RCT results will be submitted for publication to international, peer-reviewed journals, regardless of whether the results are positive, negative, or inconclusive with regard to the research aims and questions. Authorship eligibility will be based on the reviews and contributions among researchers. An annual project report will be submitted to the HREC. Any critical protocol amendments will be reported to the HREC in the annual report, updated in the Australian New Zealand Clinical Trials registry, and communicated in the preliminary RCT result reports.

## Results

This study was funded by the e-ASIA Joint Research Program in November 2020, with funding from the National Health and Medical Research Council (Australia), the Ministry of Research and Technology/National Research and Innovation Agency (Indonesia), Health Research Council (New Zealand), and the Ministry of Science and Technology (Vietnam). The study brings together complementary expertise across multiple disciplines of 39 senior, early/midcareer and predoctoral researchers in the aforementioned 4 e-DiVA countries and NGO partners including DA, ALZI, Alzheimers New Zealand, Alzheimer’s Disease International, and the WHO.

The WHO iSupport manual in English has been translated to Bahasa and Vietnamese, and the adaptation of these translated versions is being conducted prior to the production of video clips as content for the iSupport VA. From November 2021 to June 2022, the iSupport VA will be developed. The iSupport VA will enhance WHO iSupport web-based program by providing real-time supports for carers with additional interaction options of voice command and video instruction to make it more user-friendly, quicker, and easier to learn. The VA will then be tested by carers and health care professionals in 6 months from July 2022 to December 2022, and their feedback will be considered in an iterative process of refining the VA. From January to July 2023, we will enroll 140 dementia carers (20 carers per iSupport VA version) for the pilot RCT and follow them up for 6 months. This study is expected to conclude in June 2024.

## Discussion

### Expected Outcomes

Family carers of people with dementia face many challenges. Approximately 40% of family carers of people with dementia have clinically significant depression or anxiety [30]. When compared to the general population and carers of people with other chronic diseases, dementia carers have worse mental and physical health, more absences from work, and lower quality of life [31,32]. Carer burden, psychological distress, and perceived inability to provide care predict care recipient’s institutionalization [33]. Educating, upskilling, and supporting carers to reduce the burden of care and improve their mental health and well-being through low-cost and sustainable nonpharmacological intervention has become one of the 7 priority areas of action in the WHO Global Action Plan on dementia [10].

Dementia carers who are supported in their caring role benefit from support, as does the person with dementia [34]. Most effective carer interventions are multimodal, incorporating education, skill-building to manage atypical behaviors, stress reduction, and referral to community resources [35]. One of the most comprehensive multi-component carer intervention is the REACH (Resources for Enhancing Alzheimer’s Caregiver Health) model [20]. The REACH model was used in Vietnam with preliminary findings showing significant improvement for carer mental health and caregiving burden [22,36]. However, this model is resource-intensive with interventionists going to carers’ home to deliver the intervention over 3 months. Additionally, while face-to-face interventions have demonstrated positive effects on carer mental health, many carers cannot attend support programs owing to lack of transport, finances, or being unable to leave the person for whom they provide care [37].

Carer interventions delivered on the internet via an app could be an effective solution to overcome these accessibility barriers and are a viable option in crises such as the COVID-19 pandemic when social restrictive policies are in place. Internet-based approaches may be perceived more acceptable because carers can access them at their own convenience and expense [11]. Via digital health solutions via smartphones, tablets, and computers, carers can learn in their own language and cultural context about dementia care [38]. Smartphone use is exponentially increasing in the APR; by 2025, 83% of the population (2.7 billion people) will be mobile internet users, thus making smartphone digital media a crucial platform to increase community education and understanding [39].

Over 2 million people have dementia in the aforementioned 4 countries in this study—a number that will triple by 2050. The vast majority live at home, are cared for by family members, and are mainly women. Recognizing the effects of long-term dementia care on carers’ time, energy, income, health, and well-being, the e-DiVA study will test a digital solution to support dementia carers. Using co-design ensures iSupport VA’s user friendliness and trustworthiness, the study’s diverse settings ensure the cultural adaptability of iSupport VA across high-, middle-, and low-income settings. The COVID-19 pandemic has underscored the importance of and need for digital innovation for people with dementia and their carers. This study offers a solution to ensure quality care for people with dementia, no matter their location, income, or crises facing them.

To our knowledge, this is the first study using information technology solutions not only to adapt the WHO iSupport web-based program but also to enhance it by providing real-time support to carers with additional interaction options of voice command and video instruction to make it more user-friendly, quicker, and easier to learn. This is an important innovation, which will assist those with limited digital and general literacy to access information, although it will not be relevant for those carers who do not have access to the internet.

The e-DiVA study builds on our team’s prior work and expertise, strengthens and expands existing collaborations between investigators in high-income countries (Australia and the United States) and Vietnam. These include our pilot iSupport adaptation study in Australia [14] and other studies to develop Vietnam’s National Dementia Plan [40] and to support Vietnamese dementia family carers [41]. This project proposal keeps laying the continual foundation for extramural support for both research and capacity-building. The proposal directly responds to the priorities of our collaborating countries [40,42-44] and will create a multinational partnership between the NGO partners (ie, national Alzheimer/dementia associations, Alzheimer’s Disease International, and the WHO) and researchers to optimize support for dementia carers. Our commitment to ongoing co-design and stakeholder engagement in every step of our research, along with our commitment to building capacity in our stakeholders and locally based researchers will support the successful translation and ownership of iSupport VA by the local Alzheimer and dementia associations. By the end of the project, we aim to have research-ready, stakeholder engaged, local champions to enable the final translation of iSupport VA nationally, as well as its ongoing development and increasing chances of having a longer-term impact, beyond the life of the project.

### Trial Status

The trial is ongoing. Participants are not yet being enrolled. The protocol date and version are July 10, 2021, and 1.0, respectively. The trial has been registered on Australian New Zealand Clinical Trials Registry (identifier ACTRN12621001452886).

## Appendix

Multimedia Appendix 1 Peer-reviewer report from the Health Research Council of New Zealand.

## Abbreviations

ALZI: Alzheimer’s Indonesia
APR: Asia-Pacific Region
CALD: culturally and linguistically diverse
DA: Dementia Australia
HIC: high-income country
iSupport VA: iSupport virtual assistant
LMIC: low- and middle-income country
NARI: National Ageing Research Institute
NGO: nongovernment organization
NHMRC: National Health and Medical Research Council
PSS: Perceived Stress Scale
RCT: randomized controlled trial
REACH: Resources for Enhancing Alzheimer’s Caregiver Health
UTAUT: Unified Theory of Acceptance and Use of Technology
WHO: World Health Organization
