# Supplementary material for: Empowering Dementia Carers With an iSupport Virtual Assistant (e-DiVA) in Asia-Pacific Regional Countries: Protocol for a Pilot Multisite Randomized Controlled Trial
Source: JMIR Res Protoc. 2021 Nov 16;10(11):e33572. doi: 10.2196/33572 (PMC8663455; doi:10.2196/33572)
Supplement: Multimedia Appendix 1 [file resprot_v10i11e33572_app1.pdf]

Applicant peer review report

Reviewer # 51

Proposal details

Title	e-DiVA (empowering Dementia Carers with an iSupport Virtual Assistant)
First named investigator	Dr Sarah Cullum (The University of Auckland; Counties Manukau District Health Board)

Health Significance

The applicants have demonstrated the significance of enhancing education and training for family caregivers of persons living with dementia. The proposed multi-year and multi-country study will advance the knowledge relevant to health outcomes for caregivers.

Scientific Merit

The applicants made a strong case for smartphone app solutions to help family caregivers during COVID-19. The applicants included clear exclusion criteria relevant to skill level in using internet applications and internet connectivity. The hypotheses are clearly written, as are the outcome measures. One area that could strengthen the application is a brief overview of the modules within the WHO iSupport intervention and references to existing peer-reviewed publications on the intervention itself.

Design and Methods

The design is strong, with clear inclusion/exclusion criteria. One of the major strengths is the co-design with stakeholders in each country and cultural group. There were no power calculations listed, but the sample size listed for both intervention and control groups is sufficient. It would also be helpful to include an analysis by gender and the various CALD groups (or make that more explicit in the proposal).

Expertise of Research Team

This is a very strong group of researchers, who have access to other dementia care scholars if needed. One of the strengths of the proposal is incorporating a skills and capability audit of the research team and partners. Another strength is the inclusion of senior and early career researchers.

Quality of the Partnership

This is an excellent proposal with a strong team of researchers and community partners and includes a clear plan and role of all involved.

General comments

Applicant peer review report

Reviewer # 52

Proposal details

Title	e-DiVA (empowering Dementia Carers with an iSupport Virtual Assistant)
First named investigator	Dr Sarah Cullum (The University of Auckland; Counties Manukau District Health Board)

Health Significance

The proposed research (a) translates the existing WHO iSupport to local languages, and culturally and contextually adapt it to fit the health and social care systems of Australia, Indonesia, New Zealand and Vietnam; (b) develops an iSupport Virtual Assistant smart device app; and (c) ascertains the effects of the iSupport Virtual Assistant on stress, psychological distress and quality of life for informal carers of people living with dementia via a pilot randomised controlled trial. The applicants have demonstrated that the proposed research addresses a significant health issue, has the potential to advance knowledge relevant to health, and that it may contribute to improved health or health outcomes.

Scientific Merit

The rationale for the proposed study is presented well. The aims of the proposed study are clear where they focus on improving mental health and well-being of carers of people with dementia via the adaption of the WHO ‘iSupport for Dementia’, which is a self-learning online skills training and support program for informal dementia carers, for use in different countries where it is more user-friendly with real time support. Study hypotheses, while only implied in the research proposal, are acceptable. Original findings are expected from the proposed study.

Design and Methods

The study design is adequate. However, there are either insufficient detail for parts of the method and proposed analyses, or the study would benefit significantly by improvements in a one or more of these areas:

- Information regarding the translation of iSupport content for local adaption. There is some brief discussion about submission to WHO for quality assurance and the use of focus groups to assess comprehensibility and acceptability of the iSupport. Further information is needed on the validation of iSupport content (i.e. more details on these processes and the analysis to be undertaken etc.).

- Justification of sample size for RCT (why 20 participants per iSupport VA version given the three outcomes - stress, psychological distress and quality of life).

- Clarity on why the waitlist control group will receive access to the iSupport VA three months after randomisation when the intervention group will be using the iSupport VA for six months.

- Details on outcomes measures. Further information on the outcomes measures (i.e. what are the measures, how they measure and why these measures are appropriate) could be provided.

- How will missing data be managed?

- Clarity on data analysis (e.g. what are the expected covariates if any?)

Expertise of Research Team

The research team collectively have the academic qualifications, topic based knowledge, and experience to undertake the proposed research. They have a track record of publication in peer reviewed scientific journals and other professional publications, and/or experience in disseminating research. However, it does not appeared or is unclear whether the lead CIs from each of the four countries has worked together previously and/or experienced in leading / being involved in international collaborations.

Quality of the Partnership

The partnership detailed in this application does provide opportunities for contributions to existing research, training and development of New Zealand researchers and/or collaborative research in this area. The quality of the partnership could be strengthened with a discussion on how early career researchers and pre-doctoral researchers will benefit from this international collaborative research project (e.g. mentorship and potential international research experience).

General comments

The total cost of this project (~1.7 million USD) appears steep for the projected project outcomes. The NZ component seems reasonable at ~$200,000 USD. At minimum, the significance of the research study beyond the proposed project itself should be strengthened to better justified the budget costs.

Applicant peer review report

Reviewer # 55

Proposal details

Title	e-DiVA (empowering Dementia Carers with an iSupport Virtual Assistant)
First named investigator	Dr Sarah Cullum (The University of Auckland; Counties Manukau District Health Board)

Health Significance

The mental health of carers is a health priority.Prevention of mental ill health is better than treatment.If effective, a digital intervention is sustainable, relatively inexpensive.

Scientific Merit

This is an excellent team and my confidence in their capability to deliver this proposal is high.

Design and Methods

A very similar study design is to be implemented in different contexts. This means that its suitability for each one may be compromised in favour of uniformity.

Expertise of Research Team

This is a huge and diverse team. Its scale is its weakness, for example there is scope here for competing egos and global pandemics to de-rail the careful plans. Equally important therefore is the co-ordination of the study.See my general comments.

Quality of the Partnership

This is an exciting network that will enrich NZ research links.

General comments

My main concern about this mammoth programme of work is that the documents I have seen do not explain how it will be co-ordinated and managed.It appears that this is left to the 'international advisory board'.I would like to know more about the overall project admin function: where is it based, who is accountable, what are the structures and systems for managing data, issuing consortium agreements and holding researchers to account? I would also like some assurance that there is a separate budget line for overall project management, co-ordination and troubleshooting.

I would also like to point out that the initial workshop outline includes training on 'understanding dementia'.Although these are experiential workshops, I wonder why they have taken priority over 'understanding carers', who are the focus of this

research.In any educational effort, we need to strike a balance between available online learning and those things which need to be encountered face to face.There is a wealth of free, online learning about dementia.A workshop of this kind would be an invaluable opportunity to listen first-hand to the experiences of dementia carers.

Applicant peer review report

Reviewer # 67

Proposal details

Title	e-DiVA (empowering Dementia Carers with an iSupport Virtual Assistant)
First named investigator	Dr Sarah Cullum (The University of Auckland; Counties Manukau District Health Board)

Health Significance

As noted in the application, dementia is a significant and growing global health issue. The majority of care for people with dementia is undertaken by unpaid family carers who may experience health and economic impacts from their caring. It is vital that family carers are well supported for the sake of their own health and wellbeing; in order to support quality care for people with dementia; and to reduce the impacts on health systems of alternative care for people with dementia.

For Aotearoa New Zealand, this project will develop a culturally adapted version of the WHO iSupport for Dementia in te reo Māori. This is important because with an ageing Māori population, the number of Māori with dementia is expected to grow, and with it, the stress on their family carers.

Scientific Merit

The rationale for the project is clear and it addresses a significant health issue in the collaborating countries and an identified priority of their national dementia plans. The project will take an existing WHO resource (iSupport for Dementia) and adapt it culturally and linguistically for seven population groups within four Asia-Pacific countries. There is a WHO adaptation and implementation guide for doing this type of work and the project has the support of the WHO iSupport team who will assist with training at the initial project workshop and provide ongoing quality assurance.

The proposed pilot RCT will appropriately test the feasibility, acceptability and preliminary efficacy of the app with both quantitative and qualitative methods, providing the basis for a future full RCT to test the effectiveness of this carer support programme.

Design and Methods

The project will adapt an existing WHO resource for dementia carers. In all four countries, this will be undertaken through a co-design process with local carers, NGOs and professionals. Although the proposed resource adaptation work will be done in parallel in the four countries, there are appropriate methodological variations for the different contexts e.g. translation then adaptation of the resource in Vietnam and Indonesia but in New Zealand, adaptation before translation into te reo Māori.

The pilot RCT is appropriately designed to provide initial testing of the usefulness of the iSupport resource and prepare for a full RCT.

I do have two concerns around the design. The first is the sole focus on developing a resource requiring access to the internet and familiarity with using websites or apps. I accept Smartphone use will continue to grow worldwide, including among older people (who will form a significant proportion of carers for people with dementia) but there will still be a group without access to these technologies who may then be excluded from getting much-needed support. Could the researchers collect data on recruitment to the pilot RCT which records how many people are approached who decline to participate because they do not have technology access? (This will only be possible where a clinician will be directly advising carers about the study, in Indonesia, New Zealand and Vietnam and will be most useful in Vietnam where a clinician approach

appears to be the only recruitment method.)

My second concern is the lack of an Aboriginal or Torres Strait Islander group in the Australian part of the research, given the prevalence of dementia in this group is significantly higher than the general Australian population and they are known to experience poorer health outcomes than other Australians (Radford, K., Lavrencic, L. M., Delbaere, K., Draper, B., Cumming, R., Daylight, G., . . . Broe, G. A. (2019). Factors Associated with the High Prevalence of Dementia in Older Aboriginal Australians. Journal of Alzheimer's disease: JAD, 70(s1), S75-S85. doi:10.3233/JAD-180573). These groups should be high priority for further development of the iSupport VA and any future RCT.

There are three proposed in-person meetings during the project: an initial, five-day, all-of-team training workshop in Australia; a meeting in New Zealand halfway through the project to review the work and plan the pilot RCT; and a final close-out workshop in Australia. Page 18 notes these are subject to international travel being permitted and refers to a Risks section, but this seems to be missing from the proposal. How will the researchers manage the first training workshop in particular if it is not possible to meet in person? How will they manage other possible limitations on or interruptions to the research due to Covid19?

Expertise of Research Team

The project has a very experienced research team. The four country leads all demonstrate strong clinical and research backgrounds in relation to dementia. There is excellent disciplinary breadth among the research team. The inclusion of NGO partners in the research and plans to support their development in research capacity will increase the likely success and impact of the research.

Quality of the Partnership

The project builds on existing collaborations between the researchers from Australia and Vietnam. It also demonstrates partnership with local NGOs which will be vital for both the development and future implementation of this tool.

There is a demonstrated plan for capacity building across the research team and NGO partners, with an initial five-day, all-of-team training workshop and other support throughout the project (such as mentoring of newer researchers and joint analysis of qualitative data). The lead PI is an early career researcher. Two Masters and two PhD students will be involved in the project. There is diversity of gender and ethnicity among the research team (excepting indigenous Australians?).

General comments

Thank your for the opportunity to review this interesting proposal.

An article that may be of interest to the researchers:

Reilly, R., Stephens, J., Micklem, J., Tufanaru, C., Harfield, S., Fisher, I., . . . Ward, J. (2020). Use and uptake of web-based therapeutic interventions amongst Indigenous populations in Australia, New Zealand, the United States of America and Canada: a scoping review. Systematic Reviews, 9(1), 123. doi:10.1186/s13643-020-01374-x
